# Lens epithelium cell ferroptosis mediated by m^6^A-lncRNA and GPX4 expression in lens tissue of age-related cataract

**DOI:** 10.1186/s12886-023-03205-8

**Published:** 2023-12-18

**Authors:** Yong Wang, Pengfei Li, Congyu Wang, Sijie Bao, Siwen Wang, Guowei Zhang, Xi Zou, Jian Wu, Yu Guan, Min Ji, Huaijin Guan

**Affiliations:** 1 Eye Institute, Affiliated Hospital of Nantong University, 20 Xisi Road, Nantong, Jiangsu China; 2 Nantong University, Nantong, Jiangsu China; 3 The Second Affiliated Hospital of Nantong University and First People’s Hospital of Nantong City, Nantong, China; 4 Department of Ophthalmology, The Third People’s Hospital of Changzhou, Jiangsu, China

**Keywords:** Age-related cataract, N^6^-methyladenosine, Long noncoding RNA, Ferroptosis

## Abstract

**Background:**

In the present study, we explored the role of N^6^-methyladenosine (m^6^A) modification of long non-coding RNAs (lncRNAs) and its association with ferroptosis in lens epithelium cells (LECs) of age-related cataract (ARC).

**Methods:**

Through m^6^A RNA immunoprecipitation sequencing (m^6^A-RIP-seq) and RNA sequencing (RNA-seq), we identified m^6^A mediated and differentially expressed lncRNAs (dme-lncRNAs) in ARC patients. Based on bioinformatics analysis, we selected critical dme-lncRNAs and pathways associated with ARC formation to reveal their potential molecular mechanisms. The downregulation of glutathione peroxidase 4 (GPX4), a key component of ferroptosis, was confirmed by real-time RT-PCR (RT-qPCR) and Western blotting in age-related cortical cataract (ARCC) samples. Transmission electron microscopy was used to assess the change in mitochondrial in LECs.

**Results:**

The analysis revealed a total of 11,193 m^6^A peaks within lncRNAs, among which 7043 were enriched and 4150 were depleted. Among those, lncRNA ENST00000586817(upstream of the GPX4 gene) was not only significantly upregulated in the LECs of ARCC but also potentially augmented the expression of GPX4 through a cis mechanism. The expression of m^6^A-modified lncRNA (ENST00000586817) was correlated with that of GPX4 and was downregulated in ARC patients. The TEM results indicated significant mitochondrial changes in ARCC samples. GPX4 downregulation enhanced LEC ferroptosis and decreased viability via RSL3 in SRA01/04 cells.

**Conclusions:**

Our results provide insight into the potential function of m^6^A-modified lncRNAs. M^6^A-modified lncRNA ENST00000586817 might regulate the expression of GPX4 by a cis mechanism and be implicated in ferroptosis in ARCs.

**Supplementary Information:**

The online version contains supplementary material available at 10.1186/s12886-023-03205-8.

## Background

Age-related cataract is the predominant cause of blindness and visual impairment worldwide, and there is no consistent conclusion regarding its aetiology [1]. It was documented that oxidative stress, DNA damage repair, response to apoptosis, autophagy, and ferroptosis are factors involved in ARC pathogenesis [2–7]. Ferroptosis, a novel nonapoptotic form of oxidative cell death, is mainly induced by iron-dependent and lipid peroxidation [8]. The lipid hydro peroxidase glutathione peroxidase 4 (GPX4) translates lipid hydroperoxides into lipid alcohols and suppresses the iron ion (Fe^2+^)-dependent formation of toxic lipid reactive oxygen species (ROS) [9]. Moreover, the vital role of GPX4 in cataract formation has been confirmed, due to the rapid development of lens opacity in GPX4 knockout mice [10, 11]. Therefore, GPX4, as the central regulator of ferroptosis, might be crucial to the maintenance of lens transparency.

Previous studies have clarified that the expression of oxidative damage repair genes could be regulated by long noncoding RNAs (lncRNAs) [12–14]. LncRNAs can regulate targeted gene expression at the epigenetic level by interacting with mRNAs [13], microRNAs (miRNAs) [14], or proteins [15]. Moreover, our previous studies have demonstrated that several lncRNAs potentially exert an influence on ARC formation by different mechanisms [12–14]. However, many previous studies primarily focused on the regulation of target genes by lncRNAs, with less focus on the mechanisms regulating lncRNAs expression itself. Recently, N^6^-methyladenosine (m^6^A) has been identified as a structural alteration affecting lncRNA expression [16].

M^6^A is the most abundant epigenetic modification within lncRNAs in eukaryotes and plays an important role in RNA biology [17]. The modification can be regulated by multiple enzymes including m^6^A methyltransferases (METTL3, METTL14, and WTAP), demethylases (FTO and ALKBH5), and m^6^A-binding proteins (YTHDF1-3 and YTHDC1-2) [18]. Our study indicated that the altered expression of ALKBH5 could contribute to changes in circRNA m^6^A modifications in LECs [6]. Numerous studies have revealed that m^6^A modifications can regulate the expression of m^6^A-labelled lncRNAs [19–21]. Even so, research on the m^6^A modification of lncRNAs is still lacking and the roles of RNA modification in ARC formation remain unknown.

Herein, we performed genome-wide altered m^6^A-tagged lncRNA screening in LECs from age-related cortical cataracts (ARCCs). Possible functions of m^6^A-modified lncRNAs were predicted by Gene Ontology (GO) annotation and Kyoto Encyclopedia of Genes and Genomes (KEGG) pathway enrichment analysis. The data suggested that the m^6^A-modified lncRNA ENST00000586817 might be involved in the development of ARC by *cis*-targeted genes to regulate ferroptosis-related GPX4 in LECs.

## Methods

### Study participants

ARCC patients were recruited in this study and the Lens Opacities Classification III (LOCS III) system was used for the classification of disease severity [22]. In addition, age-matched individuals without cataracts were removed from vitreoretinal diseases and selected as controls. The inclusion and exclusion criteria for the selection of the participants in the current study were identical to our previous research [6]. After the abovementioned screening, 19 patients with ARCC, and 19 control patients were finally included. The basic demographic data of all participants are listed in Table 1.

**Table 1:** The grade of lens opacity and identification codes of controls and ARCCs

Controls					ARCCs			
Samples	Sex	Age(y)	LOCSIII		Samples	Sex	Age(y)	LOCSIII
No.1	Male	61	C0N1P0		No.1	Male	59	C3N1P1
No.2	Male	56	C0N1P0		No.2	Male	64	C3N1P1
No.3	Male	58	C0N1P0		No.3	Male	62	C3N1P1
No.4	Male	54	C0N1P1		No.4	Male	67	C3N1P1
No.5	Male	55	C0N1P1		No.5	Male	59	C3N1P1
No.6	Male	57	C0N1P1		No.6	Male	64	C3N1P1
No.7	Male	58	C0N1P1		No.7	Male	63	C3N1P1
No.8	Male	53	C0N0P1		No.8	Male	65	C4N1P2
No.9	Male	50	C1N1P0		No.9	Female	59	C3N1P1
No.10	Male	62	C1N0P1		No.10	Female	58	C3N0P1
No.11	Male	58	C1N1P0		No.11	Female	59	C3N1P1
No.12	Female	62	C0N1P1		No.12	Female	55	C4N1P2
No.13	Female	65	C0N1P1		No.13	Female	65	C3N1P1
No.14	Female	68	C1N1P1		No.14	Female	68	C4N1P1
No.15	Female	59	C0N0P1		No.15	Female	69	C3N2P1
No.16	Female	57	C1N1P1		No.16	Female	61	C4N1P1
No.17	Female	69	C1N0P1		No.17	Female	56	C3N1P1
No.18	Female	65	C0N1P1		No.18	Female	54	C4N2P1
No.19	Female	54	C0N0P1		No.19	Female	58	C3N1P1

### RNA extraction and MeRIP-seq

Using TRIzol reagent (Invitrogen), we isolated total RNA from LECs. The method was performed as reported in our previous study [6]. The MeRIP-seq method was used to measure m^6^A-methylated RNA, supported by Cloudseq Biotech Inc. (Shanghai, China). In brief, m^6^A immunoprecipitation was conducted with the GenSeqTM m^6^A-MeRIP Kit (GenSeq Inc., China) according to the manufacturer’s instructions. The protocol for MeRIP-seq assays has been described in our previous article [6].

### High-throughput sequencing data analysis

After sequencing on the Illumina HiSeq 4000 sequencer, paired-end reads were harvested and quality controlled by Q30(Figure S1-S2). In addition, Table S1 shows the alignment rates, sequencing depth, and peak metrics and Figure S3 illustrates the PCA plots and correlation analysis of different samples. The clean data were obtained through 3’ adaptor-trimming and removal of low-quality reads using Cutadapt software (v1.9.3). After that, clean data were aligned to the reference genome (UCSC HG19) HISAT2 via software (v2.0.4). Methylated sites on lncRNAs (peaks) were identified by MACS software and diffReps, which were detected by both software overlapping with exons of lncRNA were determined and prepared with custom-made scripts. Based on the source genes of differentially methylated lncRNAs, we performed GO and KEGG analysis [23].

Different m^6^A sites were identified by diffReps, with a cut-off of *p* ≤ 0.0001 and fold-change ≥ 2. Using homemade scripts, peaks were identified as overlapping with exons of lncRNA. The differential expression of mRNAs and lncRNAs between the two groups was assessed via the edgeR R package. LncRNAs with a cut-off of fold-change ≥ 2 were considered differentially expressed, while mRNAs with a cut-off of *p* value < 0.05 and fold-change ≥ 2 were considered differentially expressed. The algorithm was applied to search for lncRNA *cis*-regulated target genes was based on chromosome coordinates. Putative *cis*-acting *regulatory* DNA *elements* (*cis*-*elements*) regulate the transcription of neighbouring genes. This study defined genes located within 10 kbp upstream or downstream of lncRNAs to be *cis* regulated based on previous studies [24, 25].

### GO and pathway analysis

GO analysis and KEGG analysis were performed for functional annotation of differentially expressed mRNA and lncRNA *cis* targets. GO enrichment analysis was performed by the R ‘cluster Profiler’ package and KEGG enrichment analysis was tested on hypergeometric distribution by the R ‘hyper’ function. GO categories or pathways with a *p* value < 0.05 were considered significantly enriched.

### Quantitative RT-PCR (qRT-PCR)

The lncRNA ENST00000586817 and *GPX4* levels were analysed by RT***-***qPCR assay in LECs of ten ARCC patients and ten controls. In this research, the selected genes were validated by RT***-***qPCR [26]. The relative differences in gene expression between the two groups were expressed by using GAPDH as an internal control which was then compared to the target mRNA. The primer sequences and reverse primers used were as follows: ENST00000586817 forward primer CCACCAGCCACTGCTTCCT, reverse primer CACCCAACCTCCTACAACAACC; GPX4 forward primer GAGGCAAGACCGAAGTAAACTAC and reverse primer CCGAACTGGTTACACGGGAA; GAPDH forward primer TGAAGGTCGGAGTCAACGGATTTGGT, reverse primer CATGTGGGCCATGAGGTCC ACCAC. Relative fold expression changes were determined by the comparative CT (2^−△△CT^) method. Online software was used to design the gene-specific primer pairs (http://www.ncbi.nlm.nih.gov/tools/primer-blast/). All experiments were carried out in triplicate.

### Transmission electron microscopy

LECs were fixed with 4% glutaraldehyde for 3 h. After the glutaraldehyde was removed, the LECs were embedded in 1% agarose and mixed with 4% glutaraldehyde. Lens anterior capsule tissues were directly fixed with 4% glutaraldehyde for 4 h and then treated with 1% osmic acid for 1.5 h. Before the samples were embedded in epoxy resin, they were sequentially dehydrated in 50%, 70%, 90%, and 100% acetone three times for 15 min each. Section (70 nm thick) were cut and stained with uranyl acetate for 15 min. Finally, samples were observed with a transmission electron microscope (Hitachi, Tokyo, Japan).

### Western blot assays

Total protein was extracted from human LECs. The protocol for western blotting was been described in our previous study [6]. Samples were incubated overnight with polyclonal rabbit anti-human-GPX4 (1:1000, Abcam) and rabbit anti-GAPDH (1:6,000, Abcam) monoclonal antibodies. After being washed, binding of anti GPX4 IgG was detected with alkaline phosphatase-conjugated goat anti-rabbit IgG antibody (1:10,000; Santa Cruz, Dallas, TX, USA).

#### Cell viability assay

SRA01/04 cells were seeded onto 96-well plates and treated with DMSO and RSL3 (0.2 µM) (MedChemExpress, New Jersey, USA). After 24 h, the Cell Counting Kit-8 assay (Dojindo Laboratory, Kumamoto, Japan) was used to detect cell viability. The process was as follows, after specific treatment, 10 µl of CCK-8 solution was added to each well and then incubated for 2 h at 37 °C. Then the cell viability was calculated by detecting the absorbance at 405–450 nm using a microplate reader (BioTek, Vermont).

#### Measurement of Fe^2+^ and MDA levels

The Fe^2+^ level was measured by the FerroOrange method, and the corresponding reagents were purchased from Dojindo Molecular Technologies Company. SRA01/04 cells were seeded in 24-well plates at a density of 5 × 10^5^ cells/wells and cultured for 24 h, and then DMSO and RSL3 (0.2 µM) were added to the wells. Moreover, wells without treatments were prepared as a control group. After incubation for 24 h, the culture medium was removed, and the cells were washed with Hanks three times. When FerroOrange (1 µM, an intracellular Fe^2+^ ion probe, Ex: 543 nm, Em: 580 nm) dispersed in serum-free medium was added to each well, the cells were incubated for 30 min in a 37 °C incubator equilibrated with 95% air and 5% CO_2_. After the incubation was completed, fluorescence images of the cells were captured using a microscope (Leica, Germany).

Intracellular MDA concentrations were assessed using the Lipid Peroxidation MDA Assay Kit (Beyotime, Shanghai, China). After the indicated treatments, SRA01/04 cells were harvested and lysed in RIPA and CM lysis solution. After lysis at 4 degrees for half an hour, the tubes were centrifuged at 12 000 × g for 5 min and the supernatant was collected for subsequent experiments. This experiment strictly followed the manufacturer’s instructions.

### Statistical analysis

Paired-end readings were obtained from the Illumina HiSeq 4000 sequencer and were then subjected to Q30 quality control. After three adaptor-trimming and poor-quality read removals steps, cutadapt software (v1.9.3) was used. First, using STAR software, clean reads from input libraries were aligned to the reference genome (UCSC HG19).

Next, HISAT2 software aligned clean reads from all libraries to the reference genome (v2.0.4). By using MACS software, methylated sites on lncRNAs (peaks) were found. DiffReps was used to find the differentially methylated sites, and the source genes of the differentially methylated lncRNAs were used to conduct GO and pathway enrichment analyses. These peaks identified by both software programs overlapping with exon lncRNAs were identified and selected by homemade scripts. All of the results was expressed as the means ± SDs of experiments that were repeated at least three times. All statistical analyses were performed using SPSS software, version 25.0 (IBM SPSS, Armonk, NY, USA) and GraphPad Prism software 7.0 (GraphPad Software). One-way ANOVA and Student’s *t* test were used for statistical analyses, with *p* < 0.05 was considered to indicate statistical significanc.

## Results

### Genomic distribution of m^6^A-modified lncRNAs

**Fig. 1:**
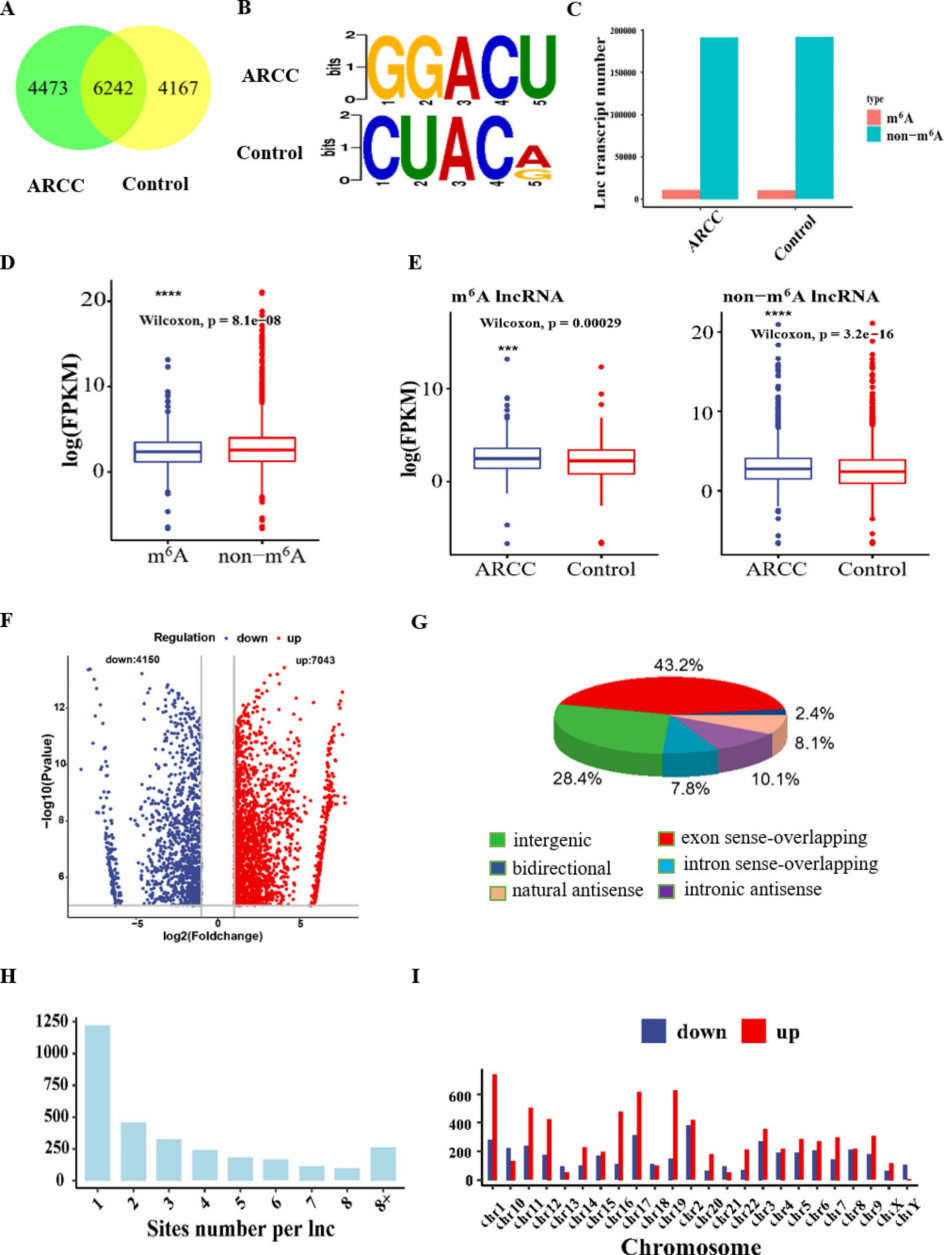
Analysis of the m^6^A-mediated lncRNAs between ARCC patients and control patients. (**A**) Venn diagram showing the numbers of lncRNA MeRIP-seq m^6^A peaks in ARCC patients and control patients. (**B**) Sequence motifs in m^6^A peaks identified from ARCC patients and control patients. (**C**) The length of m^6^A-labeled lncRNAs and non-m^6^A-labeled lncRNAs in ARCC patients and control patients. (**D**) Comparison of expression level of m^6^A-labeled lncRNAs and non-m^6^A-labeled lncRNAs in ARC. (**E**) Comparison of expression level of m^6^A-labeled and non-m^6^A-labeled lncRNAs in ARCC patients and control patients. (**F**) Volcano plots indicating differentially expressed m^6^A-labeled lncRNAs. Statistical significance compared with control patients, with fold change ≥ 1.5 and *p* < 0.05. (**G**) Distribution of different types of m^6^A-labeled lncRNAs. The percentage of each type of lncRNA identified was shown in parentheses. (**H**) Proportion of m^6^A peaks harboring different numbers of exons by per m^6^A-lncRNAs. (**I**) Chromosomal views of differentially m^6^A-labeled lncRNAs indicating the variations in their chromosomal locations. The definition of y axis is gene number

The systematic biology analysis identified a total of 14,882 m^6^A-modified lncRNAs, among which 6242 m^6^A-modified lncRNAs were shared in ARCCs and controls, 4167 m^6^A-modified lncRNAs only existed in control patients, and 4473 m^6^A-modified lncRNAs only existed in ARCCs (Fig. 1A). Motif analysis revealed consensus sequences (RRACH) in ARCCs and controls (Fig. 1B). To better define the characteristics of m^6^A-modified lncRNAs and non-m^6^A-modified lncRNAs, their lengths (Fig. 1C) and expression levels (Fig. 1D) were analysed. The distribution of lncRNA length was assessed according to the gene expression in the two groups, and it was observed that all samples showed similar. The m^6^A-modified lncRNA expression levels were lower than those of non-m^6^A-modified lncRNAs. In m^6^A-modified lncRNAs and non-m^6^A-modified lncRNAs, lncRNAs were expressed at higher levels in ARCCs than in controls (Fig. 1E).

### Distribution of m^6^A peaks in LECs

Volcano diagrams showed that 7043 hypermethylated m^6^A peaks were distributed on 1972 lncRNAs, and 4150 hypomethylated m^6^A peaks were distributed on 1049 lncRNAs (Fig. 1F). Most of the altered m^6^A modification sites were encoded by exon sense overlapping sequences (Fig. 1G) and contained only one m^6^A peak (Fig. 1H). In addition, these altered m^6^A peaks were distributed across all chromosomes, but a few chromosomes showed a higher rate of hypermethylation than hypomethylation(Fig. 1I).

### LncRNA profiling by high-throughput RNA sequencing

**Fig. 2:**
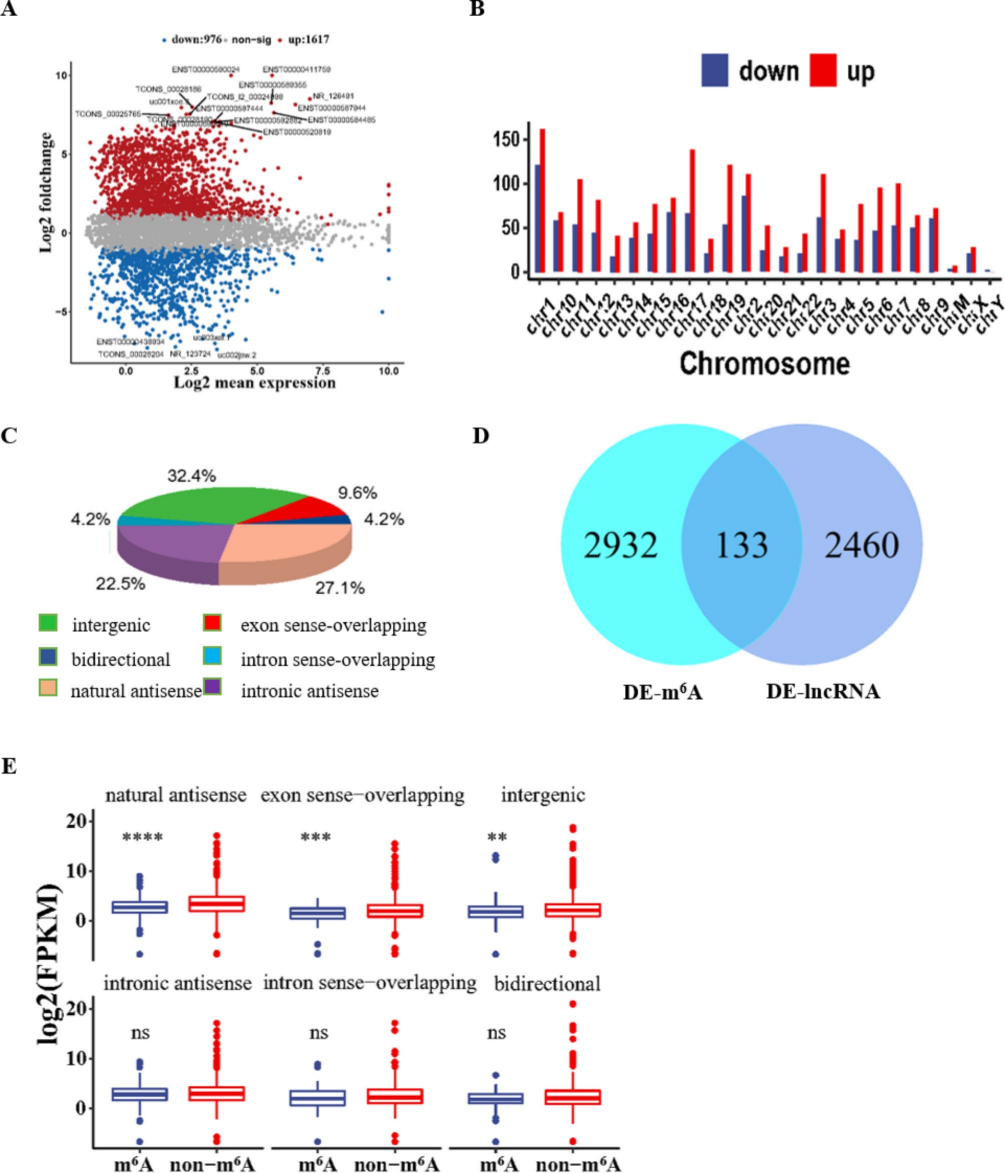
Distribution and expression of differentially lncRNAs and m^6^A-lncRNAs in MeRIP-seq and lncRNA-seq data. (**A**) The scatter plot analysis of lncRNAs with different expressions between ARCC patients and control patients. Red: upregulated lncRNAs in ARCC; blue: downregulated lncRNAs in ARCC. (**B**) Chromosomal distribution of all differentially lncRNAs. (**C**) Distribution of different types of lncRNAs. The percentage of each type of lncRNA was shown in parentheses. (**D**) Venn diagram showing overlap of m^6^A-labeled lncRNAs between in differentially m^6^A-labeled lncRNAs and differentially expressed lncRNAs. (**E**) Comparison of expression level of m^6^A-labeled lncRNAs and non-m^6^A-labeled lncRNAs in ARCC patients and control patients

LncRNAs with differential expression between the ARCCs (n = 3) and controls (n = 3) with a fold change > 2 and p value < 0.05 in the negative binomial distribution are highlighted in red (positive fold change) and blue (negative fold change). Nine hundred seventy-six lncRNAs were upregulated and 1617 lncRNAs were downregulated in the LECs of ARCCs (Fig. 2A). The top 20 lncRNAs with differential expression (DE-lncRNAs) are indicated in Fig. 2A. Furthermore, the distribution of lncRNAs in ARCC patients showed that DE-lncRNAs were transcribed from all chromosomes (Fig. 2B). Moreover, we analysed the distribution of source sites of DE-lncRNAs. The number and proportion of matching sequences of six functional components (intergenic, bidirectional, natural antisense, exon sense-overlapping, intron sense-overlapping and intronic antisense) were confirmed. Most of the matched sequences in the six samples were intergenic (Fig. 2C).

### m^6^A level and expression of lncRNAs

Then, we intersected lncRNAs with differential m^6^A methylation with the differentially expressed lncRNAs and observed the correlation between m^6^A modification and lncRNA expression changes (Fig. 2D). We also conducted a classified statistical analysis of the expression of different types of lncRNAs in m^6^A-modified lncRNAs and non-m^6^A-modified lncRNAs (Fig. 2E). Combined with the results in Fig. 1, we further explored the relation of lncRNAs expression to the different origins of lncRNAs. The expression of lncRNAs derived from intronic antisense, intron sense-overlapping and bidirectional sequences was significantly different between the ARCC group and the control group. Our results demonstrated that downregulated m^6^A-modified lncRNAs were derived from three functional coding regions.

### Construction of the ARC-related m^6^A-modified lncRNA networks via cis mechanism

**Fig. 3:**
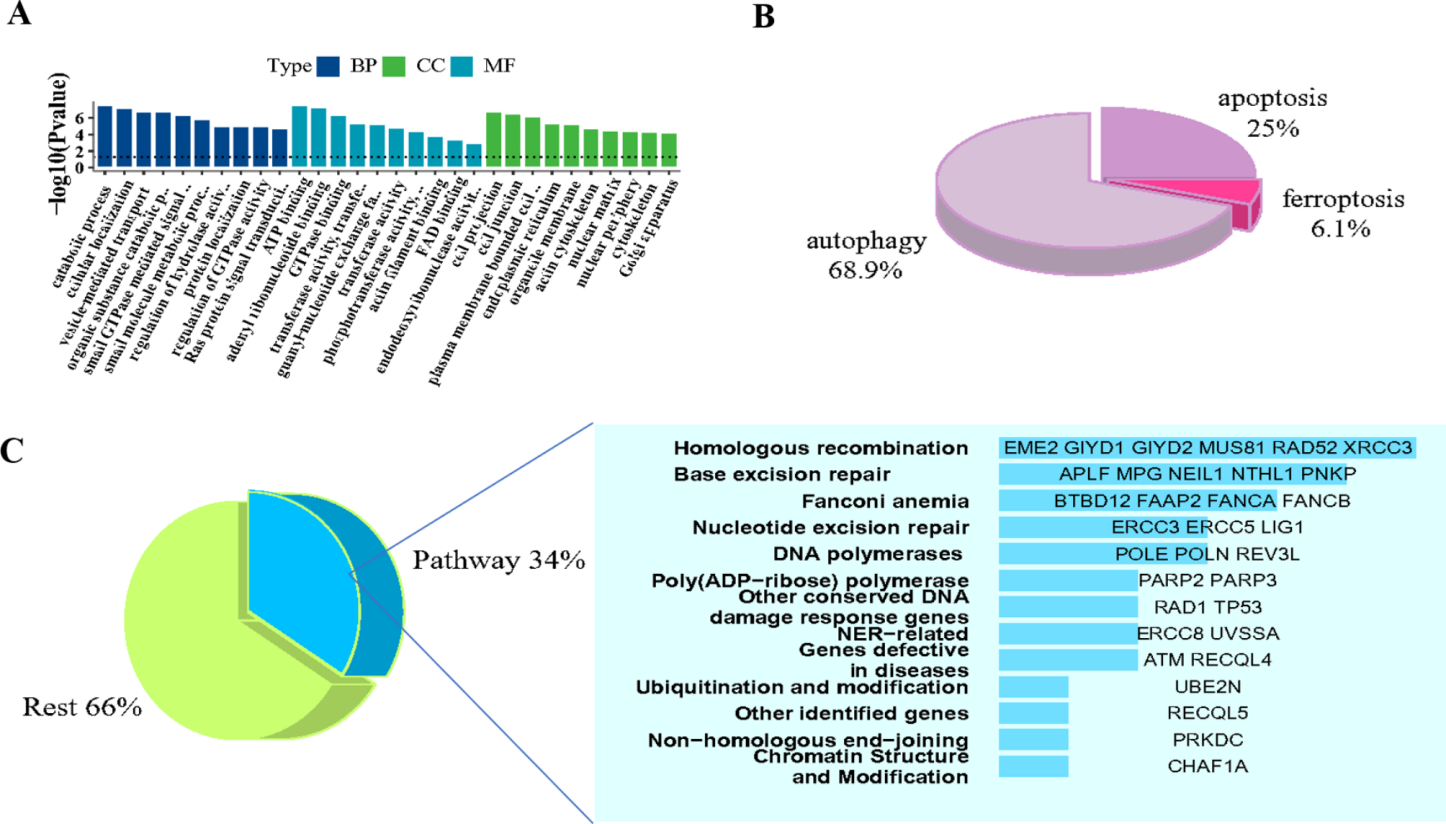
Differentially expressed lncRNAs modified by differential methylation were analyzed by the function of the target RNAs, combined with m^6^A methylation sequencing and mRNA-seq. (**A**) Enriched GO terms of host genes relevant to ARC pathogenesis. (**B**) The pie chart classify the genes involved in cell death pathway using KEGG that are controlled by lncRNA through cis mechanisms. (**C**) Use pie chart to classify the results in Figure A. The pie chart classify the genes involved in DNA repair pathway using KEGG that are controlled by lncRNA through cis mechanisms. The right bars represent the target genes of differentially expressed lncRNAs within several known DNA repair pathways (34%). The longer bar indicates more genes included. 66% represents unknown DNA repair pathways

LncRNAs not only act as sponging miRNAs, but also regulate gene expression through *cis*-targeted genes and RBP mechanisms. Hence, we constructed a network diagram of lncRNAs-mRNAs using *cis* mechanism. The algorithm was used to search for *cis*-regulated target genes via gene annotations provided by Cloudseq Biotech Inc. (Shanghai, China). Genes located within 10 kbp upstream or downstream of the lncRNAs were considered *cis*-regulated target genes. Next, we used GO analysis to assess the functional enrichment of these target genes, as shown in Fig. 3A. In our previous study, we preliminarily screened the expression of ferroptosis-related genes in ARC [6]. In addition, autophagy and apoptosis mechanisms in ARC have also been reported [6, 12, 13]. Among these genes, we mainly found that ferroptosis was significantly enriched in ARCC patients. In addition, autophagy- and apoptotic-related lncRNAs were also screened (Fig. 3B). The pie chart was used to show genes directly affected by differentially expressed lncRNAs by RNA-seq and to screen out target genes Involved in the DNA repair pathway (Fig. 3C). Table 2 shows that the lncRNAs screened through analysis of *cis*-targeted genes were correlated with DNA damage repair and cell death. Furthermore, ferroptosis was a relatively enriched death pathway among the *cis* mechanisms of lncRNAs, as shown in Table 3.

**Table 2:** Differentially m^6^A-lncRNAs and acted on target genes associated with ARC

lncRNA_id	GeneSymbol	LFC_ targetgene	methylation_ statu	Type	type
NR_026964	GPSM1	-5.635	up	autophagy	Bidirectional
NR_002612	TRIM13	5.249	down	autophagy	natural antisense
ENST00000586817	GPX4	2.668	up	ferroptosis	Intergenic
ENST00000568298	ATP6V0D1	6.056	up	autophagy	exon sense-overlapping
ENST00000560504	PIF1	5.647	up	DNA repair	exon sense-overlapping
ENST00000555140	PARP2	-5.708	up	DNA repair	exon sense-overlapping
ENST00000555140	PARP2	-5.708	up	apoptosis	exon sense-overlapping
ENST00000534664	PARP2	-5.708	up	DNA repair	exon sense-overlapping
ENST00000534664	PARP2	-5.708	up	apoptosis	exon sense-overlapping
ENST00000534495	LMNB2	-3.169	up	apoptosis	exon sense-overlapping
ENST00000481708	SMC6	5.015	down	DNA repair	exon sense-overlapping

**Table 3:** Differentially m^6^A-lncRNAs and acted on target genes associated with ferroptosis

lncRNA_id	Gene Symbol	LFC_−_ targetgene	Methylation_−_ statu	Type	Type
ENST00000586817	GPX4	2.668	up	ferroptosis	Intergenic
ENST00000571370	TP53	-3.068	up	ferroptosis	intron sense-overlapping
ENST00000467842	TF	-2.612	up	ferroptosis	exon sense-overlapping
ENST00000490521	VDAC2	-0.061	up	ferroptosis	Intergenic
ENST00000476518	ATG5	2.577	up	ferroptosis	exon sense-overlapping
ENST00000489047	ACSL6	0.716	up	ferroptosis	exon sense-overlapping
ENST00000461278	ATG7	0.216	up	ferroptosis	exon sense-overlapping
ENST00000460291	ATG7	0.216	down	ferroptosis	exon sense-overlapping
ENST00000518348	SLC39A14	2.322	down	ferroptosis	exon sense-overlapping

### GPX4 is downregulated in ARC

**Fig. 4:**
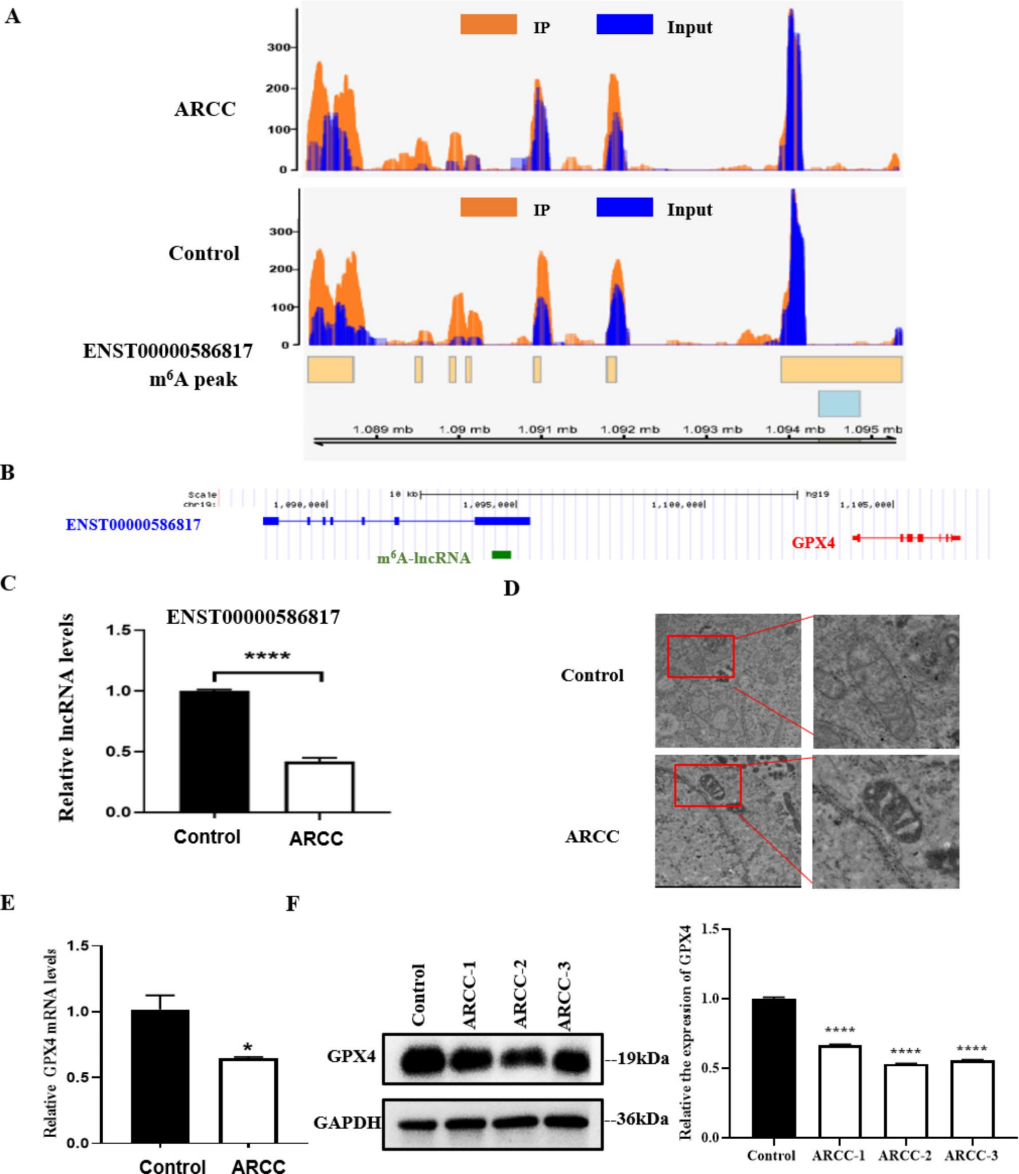
GPX4 may a target of lncRNA ENST00000586817 by *cis* mechanism and inhibit LECs ferroptosis in ARC. (**A**) MeRIP-Seq analysis on the distribution of m^6^A peak within lncRNA ENST00000586817. (**B**) Diagram showing ENST00000586817 and GPX4 locus on chromosome 19. (**C**) qRT-PCR analysis for validation the expression of DE-lncRNA ENST00000586817 in human LECs (n = 10 each). qRT-PCR data were normalized against the GAPDH level. (**D**) TEM analysis indicated that mitochondria were increased in ARC (n = 3 each). (**E**) qRT-PCR analysis was performed on the *GPX4* in human LECs (n = 10 each). qRT-PCR data were normalized against the GAPDH level. (**F**) Western blot analysis for the expression of GPX4 protein (n = 3 each). Significance value: ns, no significance, *p < 0.05, ****p < 0.0001

As shown in Table 3, GPX4 appeared in the downstream target molecules of the lncRNA-mRNA network diagrams associated with ferroptosis. According to the functional prediction, m^6^A modification upregulated m^6^A-modified lncRNAs (ENST00000586817) may regulate GPX4 expression through a cis mechanism to influence ferroptosis pathways. MeRIP-seq tracks revealed the m^6^A peak distribution in lncRNA ENST00000586817 (Fig. 4A). The data revealed that m^6^A modification was increased in ARCC. In addition, we found that the GPX4 gene was located next to lncRNA ENST00000586817 on the chromosome 19 (Fig. 4B). Then, we validated lncRNA ENST00000586817 expression in ten ARCC patients and ten control patients by qPCR. The results indicated that the expression of lncRNA ENST00000586817 decreased in ARCCs (Fig. 4C). Furthermore, we validated the decrease in GPX4 expression levels in ARCCs (Fig. 4E F). Our data suggest that decreased GPX4 might be the chief cause of enhanced LEC ferroptosis in ARC.

As shown in Fig. 4D, TEM results indicated significant mitochondrial membrane thickening and mitochondrial crista breakage and even disappearance were possibly the major ultrastructural damage type induced by the decreased GPX4 in the ARC.

### RSL3 mediated lens epithelial ferroptosis by inhibiting GPX4

Given that RSL3 is a covalent inhibitor of a central regulator of ferroptosis, GPX4, we used it to intervene in the expression of GPX4 (Figure S4 A). SRA 01/04 cells treated with 0.2 µM RSL3 did not show proliferative activity compared with untreated cells (Figure S4 B). Moreover, decreased GPX4 expression leads to lipid ROS generation, and Fe^2+^ induces ferroptosis (Figure S4 C-D), supporting the hypothesis that aberrantly expressed GPX4 promoted ferroptosis in ARC. Taken together, considering with the results of these experiments and those our m^6^A-modified lncRNA analysis, the regulatory network of m^6^A-modified lncRNA ENST00000586817-GPX4 is worthy of in-depth study.

## Discussion

Emerging evidence has demonstrated the involvement of deregulated lncRNAs in the pathogenesis of multiple ocular diseases, including ARC [12–14]. LncRNA degradation is not significantly different from mRNA degradation. Recently, increasing attention has been focused on m^6^A RNA modification which is regarded as a new epigenetic event and has been demonstrated to affect the degradation of targeted lncRNAs and participate in the progression of various age-related diseases [26–28]. However, the function of this new RNA modification of lncRNAs in ARC formation has not been characterized.

In this study, we used genome-wide profiling of m^6^A-labelled lncRNAs between ARCCs and controls. Our results showed that the expression of differentially regulated lncRNAs was comparable between the two groups. There were 1617 upregulated m^6^A-lncRNAs and 976 downregulated m^6^A-modified lncRNAs in ARCCs. Our results suggest that the modification of m^6^A-modified lncRNAs is significantly upregulated in ARC LECs. M^6^A modification is regulated by RNA methyltransferases, which have been named “writer”, and can be removed by RNA demethylases, including FTO and ALKBH5 [29]. In our previous study, the expression of ALKBH5 was significantly increased in LECs of ARCCs among five major methyltransferases [6]. We speculated that the altered methyltransferase expression induced the m^6^A RNA modification change and contributed to the downregulation of lncRNAs. Then, we compared the expression of m^6^A modified lncRNAs, and non-m^6^A-modified lncRNAs between ARCCs and controls. Overall, the m^6^A-levels of lncRNAs were negatively correlated with the expression levels of lncRNAs. In ARCC, m^6^A-levels of lncRNAs were negatively correlated with the expression levels of lncRNAs. The analysed data showed that the expression levels of m^6^A-labelled lncRNAs and non-m^6^A-labelled lncRNAs in the ARCC patients were all higher than those in controls. In addition, these data underscored the dynamic process regulating the m^6^A methylation levels on lncRNAs and the expression level of m^6^A-labelled lncRNAs in ARCCs.

M^6^A modifications of lncRNAs is reversible, and which may act as a switch to control lncRNA functionality and further impact cellular function by various underlying mechanisms associated with human diseases. Many previous studies have found that lncRNAs can regulate gene expression to mediate ARC formation through multiple mechanisms [12]. One mechanism is functioning as a competing endogenous (ce)RNA to bind to miRNA. As shown in our study, lnc-PLCD3 regulated PLCD3 expression by sponging miR-224-5p in ARC as a ceRNA [14]. lncRNA H19 could be a useful prognostic marker of early ARC and a promising therapeutic target for ARC treatment [12]. Antisense lncRNAs can downregulate gene expression by binding to target genes. For example, RNA GPX3-as decreased the apoptosis of LECs by directly promoting GPX3 expression [13]. In addition to the above mechanisms, lncRNAs function in a wide range of biological processes and can regulate gene expression in *cis* mechanisms [30]. Although that most efforts have concentrated on individual lncRNAs, researchers have attempted to classify intergenic lncRNAs based on their genomic positions relative to protein coding loci and have found a remarkable pattern of *cis* regulation by divergent lncRNAs at adjacent sites [31]. One classic *cis*-acting intergenic lncRNA Haunt (HOXA upstream noncoding transcript) is located on 40 kb upstream of the HOXA cluster [32, 33]. Interestingly, the lncRNA and its genomic locus exert opposing influences in regulating the same target genes. Through the Haunt DNA locus, which includes potential HOXA enhancers, Haunt RNA acts in *cis* to prevent abnormal high-level transcription, thus promoting fine-tuned expression of HOXA genes during embryonic stem cell differentiation [32]. The intergenic lincRNA Peril provides another example of how a genomic lncRNA sequence can serve as a *cis* enhancer for nearby transcription [34]. The expression of Peril and nearby SOX2 in mESCs was abolished via genomic deletion of a superenhancer upstream of the first intron of Peril, contributing to a global expression change and reduced proliferation of mESCs [34]. Thus, intergenic lncRNA can regulate nearby gene expression and involve in a number of biological processes [31]. Overall, *cis* regulation of nearby transcription by intergenic lncRNAs plays an important role in regulation of gene expression. Here we aimed to identify lncRNA regulation of gene expression through *cis* mechanisms. In this study, we aimed to identify lncRNA regulation of gene expression through *cis* mechanisms. Combined with analysis of DE-m^6^A-lncRNAs, DE-lncRNAs, DE-mRNAs and the intersection of genes associated with ARC pathogenesis including DNA damage repairs and cell death pathways, we enriched the m^6^A-lncRNA-mRNA network related to the mechanisms of ARC. Among this, GPX4 appeared in the downstream target molecule in the lncRNA-mRNA network diagrams. Based on the location of the intergenic lncRNA ENST000000586817 in the genome, we found that the transcript of GPX4 is adjacent. We therefore surmise that downregulated m^6^A-modified lncRNA (ENST00000586817) might regulate GPX4 expression in ferroptosis networks.

Ferroptosis is a recently discovered iron-dependent cell death process characterized by the abnormal accumulation of iron-dependent oxidative damage products in cells. In contrast to apoptosis, necroptosis, and other forms of non-apoptotic cell death, it is unique in the central involvement of iron-dependent lipid ROS accumulation and can be triggered by small molecules that block GSH synthesis or GPX4 activity. Ferroptosis is manifested by an increase in Fe^2+^ and lipid peroxidation [35]. Many studies have explored the relationship between iron concentration and cataract formation. For instance, the levels of iron in cataractous human lenses were increased compared to those in clear lenses [36, 37]. These increased iron contents could be identified as “redox available” in the cataractous lens and might be potentially cataractogenic [38]. In addition, the experimental findings of several groups support that lipid peroxidation could be one of the initial mechanisms of cataractogenesis [39–42]. In this study, we found that downregulation of the expression of GPX4 in SRA01/04 cells using the GPX4 inhibitor RSL3 led to higher more lipid ROS and Fe^2+^ levels, which induced ferroptosis. Cell viability was also significantly inhibited. In terms of epigenetics, research on the occurrence and regulation of ferroptosis is still in its infancy. Another study screened for transcript levels of some key ferroptosis-related genes in ARCs by transcripyome sequencing analysis, but the upstream regulatory mechanism has not yet been studied [7]. Therefore, our study aimed to enrich the knowledge on ferroptosis epigenetics, and provides a new direction for understanding the pathogenesis of ARCs.

Although, this study focused on m^6^A-lmodified lncRNA networks, some limitations still exist. Based on bioinformatics analysis, there is a lack of experiments to validate these results in this study. Another limitation is the small sample size and future larger sample study was required to validate our results. Although, our study offers a new perspective for the lncRNA regulation through the m^6^A-modification for further studies with larger sample sizaes are exploring the mechanisms and functions of m^6^A-lncRNAs for ARC. Moreever, FISH and nuclear- and cytoplasmic-fractionated RT-qPCR both will be needed to indicate the effects of lncRNA and GPX4 in the future experiments. Furthermore, we also need to systematically illustrate the morphological characteristics and metabolic regulation of mitochondria in the regulation of ferroptosis. Finally, experiments in related in *vivo* and in vitro models could be the future direction to certify these speculations.

## Conclusions

A significantly higher level of m^6^A modification on lncRNAs was observed in the ARC group vs. the control group. Moreover, the expression of m^6^A-tagged lncRNAs was found to be mainly decreased. Bioinformatics analyses were used to explore the potential biological functions of m^6^A-labelled lncRNAs. The data suggested that lncRNA ENST00000586817 may regulate the target gene GPX4 which is related to DNA damage and ferroptosis through cis regulation. The findings provide new insights into the mechanisms of ARC pathogenesis.

### Electronic supplementary material

Below is the link to the electronic supplementary material.

Supplementary Material 1: Figure S1. RNA Integrity and gDNA contamination test by Denaturing Agarose Gel Electrophoresis. The first three numbers in the figure correspond to the control groups and the last three numbers represent patients in the ARCC group

Supplementary Material 2: Figure S2. RNA Integrity Number(RIN) detected by Agilent 2100 RNA Nano 6000 Assay. (A-C) The three figures represent RIN within the control group. (D-F) The three figures represent RIN within the ARCC group

Supplementary Material 3: Figure S3. PCA plots and correlation analysis of 3 pairs samples. (A) PCA analysis of 3 pairs samples. (B) Correlation analysis of 3 pairs samples

Supplementary Material 4: Table S1 RNA Integrity and gDNA contamination test by Denaturing Agarose Gel Electrophoresis

Supplementary Material 5: Figure S4. The inhibition of GPX4 by RSL3 induces SRA01/04 ferroptosis. (A) Western blot analysis for the expression of GPX4 protein in SRA01/04 cells treatment by 0.2 ?M RSL3. (B) CCK8 analysis were measured the SRA01/04 cells? viability after 24h treatment with 0.2 ?M RSL3. (C) The malonaldehyde concentration were increased in group treatment with 0.2 ?M RSL3. (D) FerroOrange analysis was performed on detect the ferrous ion in SRA01/04 cells treatment by 0.2 ?M RSL3. Significance value: ns, no significance, ***p < 0.001, ****p < 0.0001

## Data Availability

The data had been submitted to gene expression omnibus (https://www.ncbi.nlm.nih.gov/geo/) (accession number, GSE153722).

## Abbreviations

ARC: age related cataract
GPX4: glutathione peroxidase 4
LncRNAs: long none-coding RNAs
miRNAs: microRNAs
m6A: N6-methyladenosine
GO: gene ontology
KEGG: kyoto encyclopedia of genes genomes
ce: competing endogenous
