# Supplementary figures and images for: Lens epithelium cell ferroptosis mediated by m6A-lncRNA and GPX4 expression in lens tissue of age-related cataract
Source: BMC Ophthalmol. 2023 Dec 18;23:514. doi: 10.1186/s12886-023-03205-8 (PMC10726616; doi:10.1186/s12886-023-03205-8)

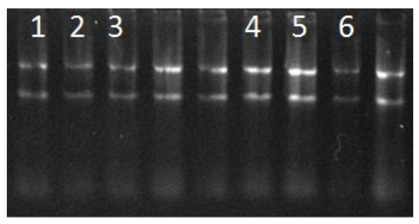

Supplement: Supplementary file 1 — Supplementary Material 1: Figure S1. RNA Integrity and gDNA contamination test by Denaturing Agarose Gel Electrophoresis. The first three numbers in the figure correspond to the control groups and the last three numbers represent patients in the ARCC group [file 12886_2023_3205_MOESM1_ESM.tif]

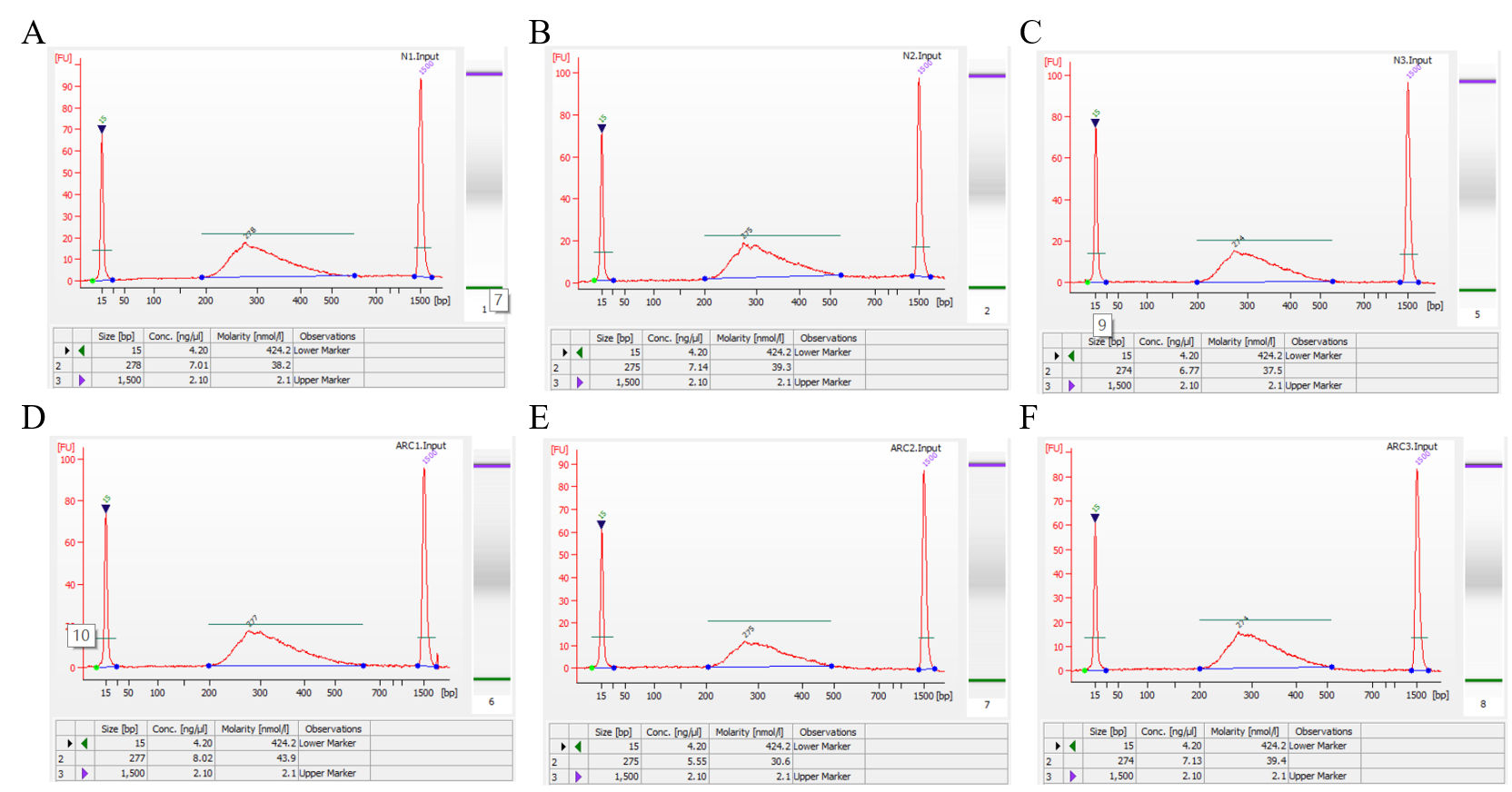

Supplement: Supplementary file 2 — Supplementary Material 2: Figure S2. RNA Integrity Number(RIN) detected by Agilent 2100 RNA Nano 6000 Assay. (A-C) The three figures represent RIN within the control group. (D-F) The three figures represent RIN within the ARCC group [file 12886_2023_3205_MOESM2_ESM.tif]

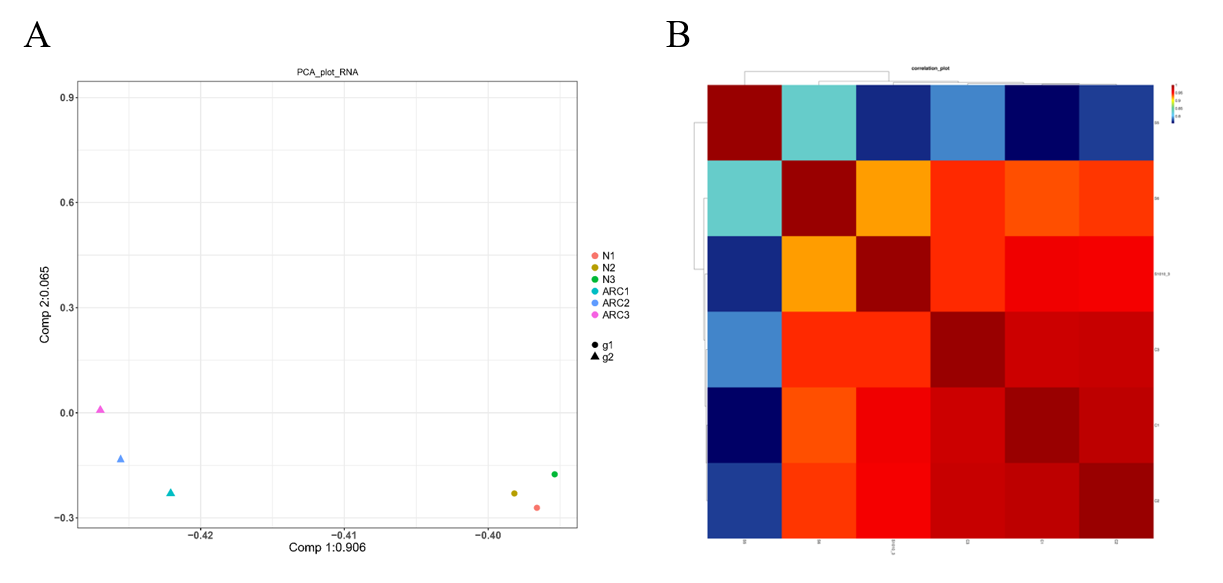

Supplement: Supplementary file 3 — Supplementary Material 3: Figure S3. PCA plots and correlation analysis of 3 pairs samples. (A) PCA analysis of 3 pairs samples. (B) Correlation analysis of 3 pairs samples [file 12886_2023_3205_MOESM3_ESM.tif]

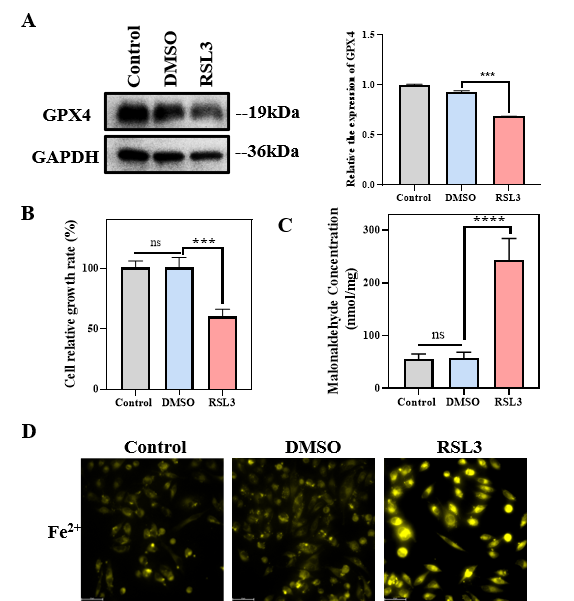

Supplement: Supplementary file 5 — Supplementary Material 5: Figure S4. The inhibition of GPX4 by RSL3 induces SRA01/04 ferroptosis. (A) Western blot analysis for the expression of GPX4 protein in SRA01/04 cells treatment by 0.2 ?M RSL3. (B) CCK8 analysis were measured the SRA01/04 cells? viability after 24h treatment with 0.2 ?M RSL3. (C) The malonaldehyde concentration were increased in group treatment with 0.2 ?M RSL3. (D) FerroOrange analysis was performed on detect the ferrous ion in SRA01/04 cells treatment by 0.2 ?M RSL3. Significance value: ns, no significance, ***p < 0.001, ****p < 0.0001 [file 12886_2023_3205_MOESM5_ESM.tif]
