# Supplementary material for: Lens epithelium cell ferroptosis mediated by m6A-lncRNA and GPX4 expression in lens tissue of age-related cataract
Source: BMC Ophthalmol. 2023 Dec 18;23:514. doi: 10.1186/s12886-023-03205-8 (PMC10726616; doi:10.1186/s12886-023-03205-8)
Supplement: Supplementary file 4 — Supplementary Material 4: Table S1 RNA Integrity and gDNA contamination test by Denaturing Agarose Gel Electrophoresis [file 12886_2023_3205_MOESM4_ESM.docx]

Table S1 RNA Integrity and gDNA contamination test by Denaturing Agarose Gel Electrophoresis.

| Sample ID | Sample Name | OD260/280 | Conc.(ng/μl) | Volume(μl) | Quantity(μg) |
| --- | --- | --- | --- | --- | --- |
| 1 | N1.Input | 1.85 | 107.68 | 9 | 0.97 |
| 2 | N2.Input | 1.82 | 98.34 | 8 | 0.79 |
| 3 | N3.Input | 1.83 | 79.88 | 8 | 0.64 |
| 4 | ARC1.Input | 1.80 | 106.54 | 8 | 0.85 |
| 5 | ARC2.Input | 1.81 | 102.77 | 9 | 0.92 |
| 6 | ARC3.Input | 1.88 | 83.51 | 8 | 0.67 |
